# How to Select Each Component of CO_2_ Electrolyzers

**DOI:** 10.1002/EXP.20240025

**Published:** 2025-08-28

**Authors:** Gyeong Ho Han, Jungmin Yoo, Juho Ha, Dong Young Hwang, Soo Young Kim, Sang Hyun Ahn

**Affiliations:** ^1^ Department of Chemical Engineering Chung‐Ang University Seoul Republic of Korea; ^2^ Department of Materials Science and Engineering Korea University Seoul Republic of Korea

**Keywords:** CO_2_ reduction reaction, CO_2_ electrolyzer, flow channel, membrane, porous transfer electrode

## Abstract

Electrochemical CO_2_ electrolyzers are increasingly recognized for their potential to convert CO_2_ into valuable chemical feedstocks, addressing critical environmental and economic challenges. Traditionally, the catalytic properties of the cathode, where CO_2_RR directly occurs, have been the main focus of research due to their control over product selectivity. More recently, however, membrane‐based electrolyzers—commonly used in fuel cells and water electrolyzers—have shown substantial potential for commercial CO_2_ reduction, offering improved scalability and efficiency. Nevertheless, the complex components in membrane‐based electrolyzers require precise optimization, as each unit directly impacts system performance and product selectivity. In this review, the structures and components of membrane‐based CO_2_ electrolyzers are systematically examined, including the electrolyzer design, flow channels, membranes, electrolytes, CO_2_ supply units, and electrodes. Recent innovations in the optimization of these components are highlighted to provide insights into advancing CO_2_RR technology toward commercially feasible applications. This approach can assist considerably in improving the CO_2_RR electrolyzer performance, thereby helping predict optimal pathways for commercial realization and guide future development.

## Graphical abstract

This review discusses how each component of a CO_2_ electrolyzer, such as membranes, electrodes, electrolytes, flow channels, and inlet CO_2_ affects system performance. By analyzing recent advances and design strategies, it highlights how optimized component selection can improve efficiency and product selectivity. These insights provide practical guidance for designing CO_2_ electrolyzers suited for industrial‐scale applications.

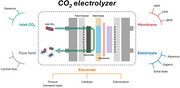

## 1 Introduction

Advances in the power generation, manufacturing, and transportation industries have accelerated greenhouse gas emissions. Consequently, the atmospheric concentration of CO_2_ is expected to reach 570 ppm by 2100, which could exacerbate adverse environmental issues such as global warming [1, 2, 3, 4]. The temporary reduction in CO_2_ emissions during the COVID‐19 pandemic demonstrates that proactive actions and close global cooperation can aid in mitigating the climate crisis [5, 6]. Previous efforts to reduce the atmospheric CO_2_ concentration were primarily focused on carbon capture and storage (CCS) technologies. However, to overcome the effectiveness‐ and sustainability‐related concerns of CCS technologies, interest in carbon capture, utilization, and storage schemes is increasing [7, 8]. Among the various CO_2_ conversion methods, the electrochemical method for converting CO_2_ into valuable chemicals and fuels is the most promising because it can be performed under mild conditions (room temperature and atmospheric pressure) and it permits storage of electricity and that of CO_2_ as liquid and gas fuels in a relatively high ratio [9]. The electrochemical CO_2_ reduction reaction (CO_2_RR) converts CO_2_ into useful products including C_1_ products including CO, HCOOH, CH_4_, and CH_3_OH, and C_2+_ products including C_2_H_4_, C_2_H_5_OH, CH_3_COOH, and C_3_H_7_OH [10]. Additionally, the use of electrochemical CO_2_RR systems allows the captured CO_2_ to be collected and recycled, enabling an effective carbon cycle. As renewable energy sources such as solar, wind, and geothermal energy are becoming increasingly more viable and cost‐effective, renewable‐energy‐based CO_2_ electrolysis is approaching large‐scale commercialization [11].

The performance of electrochemical CO_2_RR systems has been evaluated using several key metrics [12], with current density, Faradaic efficiency (FE), energy efficiency, and single‐pass conversion efficiency (SPCE) being generally considered. The current density (mA cm^−2^ or A cm^−2^) represents the amount of current flowing per unit area of the active electrode, directly indicating the reaction rate. The FE is the percentage of the total current that contributes to the formation of a specific product, reflecting the selectivity of the reaction. The energy efficiency is the ratio of the chemical energy produced to the electrical energy supplied, and serves as a crucial metric for evaluating economic viability [13]. The SPCE represents the percentage of CO_2_ converted in a single pass through the reactor and is used to evaluate the efficiency of the system and optimize the reactor design [14]. Because the CO_2_RR can yield various products, enabling high selectivity for the target compound is imperative to facilitate separation. These metrics play a crucial role in increasing the commercial and industrial feasibility of the CO_2_RR. However, the CO_2_RR exhibits a low energy efficiency because it requires a cell voltage higher than the theoretical potential. Although the interactions between the electrode and membrane, as well as the correlation between the electrode and the flow channel, have been investigated to address these issues, research on these side systems remains rudimentary [15]. Consequently, the side systems of the CO_2_RR electrolyzer should be actively studied.

CO_2_RR systems have evolved from H‐type cells to membrane electrode assembly (MEA)‐type electrolyzers and flow‐type electrolyzers. H‐type cells are primitive electrolyzers comprising two electrolyte‐filled chambers with electrodes positioned at the center of each chamber [16]. Although these cells are simple and useful for testing various catalysts, they have limitations in large‐scale applications owing to their low current density and inefficient CO_2_ gas delivery [17]. To overcome these issues, research attention shifted to MEA‐type electrolyzers, which integrate polymer electrolyte membranes and porous transfer electrodes (PTEs) to improve the inter‐electrode ion transport, thereby enhancing the current density and energy efficiency [18]. Furthermore, flow‐type electrolyzers were developed to achieve high efficiency, scalability, and effective collection of liquid products [19]. These electrolyzers continuously manage the flow of gases and liquids within the reactor, achieving a high current density and SPCE, rendering them suitable for industrial applications [20]. Additionally, the flow‐type electrolyzers have been designed to collect liquid products effectively, highlighting their superior commercial viability.

Several variables influence the operation of CO_2_RR systems. The electrolyzer components include the flow channel, PTE, and electrode [21, 22], and the external operational variables include the input CO_2_ state, flow rate, electrolyte, and pH [23, 24].

This paper reviews recent research on improving the performance metrics of CO_2_RR systems and overcoming the associated challenges. The characteristics, advantages, and limitations of each cell constituent are detailed. Furthermore, the substances produced and the catalysts used in CO_2_RR systems are explored. Specifically, membranes, electrolytes, PTEs, and flow channels are examined, and directions for future research are briefly suggested.

## 2 Important Constituents of CO_2_RR Systems

### 2.1 Electrolyzer

**FIGURE 1:**
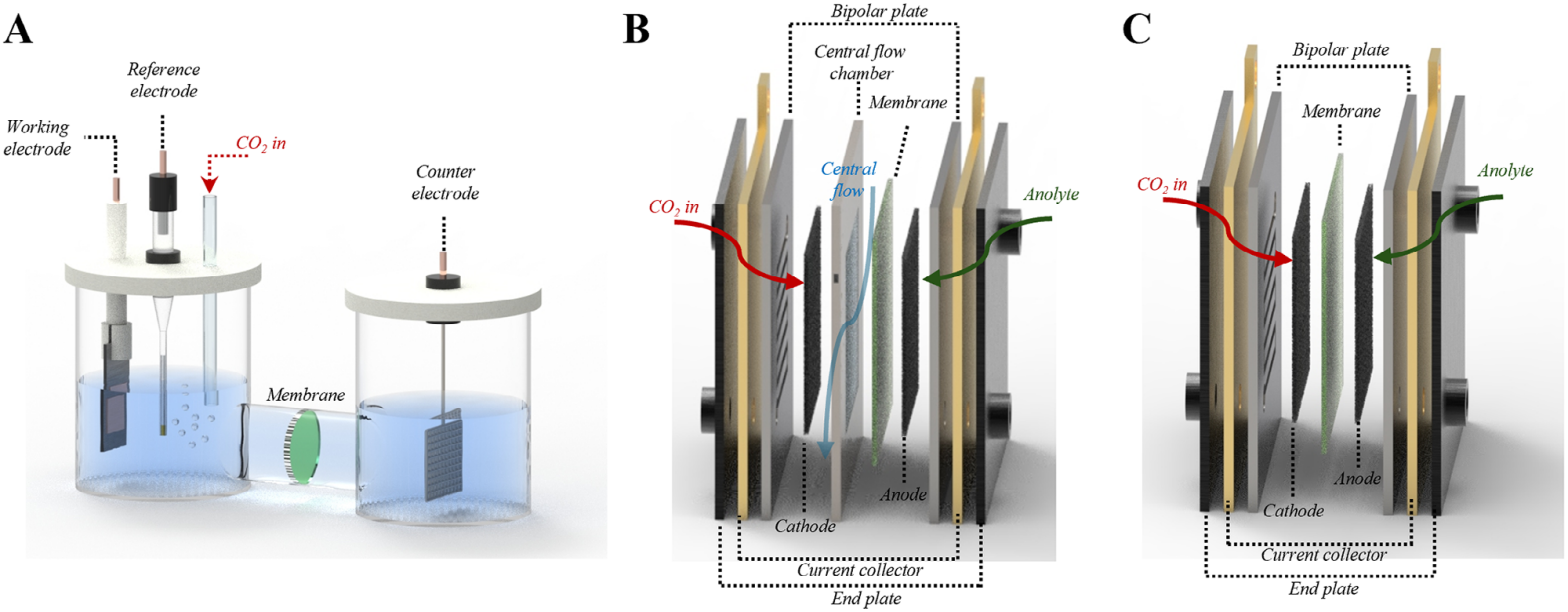
Schematic illustration of CO_2_RR electrolyzers. (A) H‐type cell, (B) flow‐type electrolyzer, and (C) MEA‐type electrolyzer.

Numerous studies have recently been conducted on the CO_2_RR, and electrolyzers have been selected depending on the purpose of the research. CO_2_RR electrolyzers are based on the single‐cell and stacked‐cell structures traditionally used in fuel cells and water electrolyzers. As mentioned earlier, CO_2_RR electrolyzers are typically categorized into: (1) H‐type cells, (2) flow‐type electrolyzers, and (3) MEA‐type electrolyzers (Figure 1 and Table 1). These electrolyzers have unique characteristics but exhibit varying shapes and structures, which can lead to different results even when studying the same catalyst or electrode. Additionally, unlike in fuel cells and water electrolyzers—in which only gas is supplied or only liquid is supplied by reactants, respectively—in CO_2_RR systems, CO_2_ gas acts as a reactant in a liquid environment; therefore, the electrolyzer must be selected considering this feature.

**TABLE 1:** Advantages and disadvantages of different types of CO_2_RR electrolyzers.

	H‐type cells	Flow‐type electrolyzers	MEA‐type electrolyzers
Advantages	Simple setup and adaptable to various materials. Useful for catalyst property studies and mechanism exploration. Allows CO_2_ saturation for initial testing.	High mass transfer efficiency with short CO_2_ diffusion. Maintain high local pH for better C_2+_ product formation. Separates gas and liquid products effectively.	Minimal ohmic resistance with zero‐gap design. High current density and scalability for industry. Excellent product separation and compact structure
Disadvantages	Low CO_2_ solubility limits performance. High ohmic losses and mass transfer limits. Inefficient for large‐scale applications.	Requires a hydrophobic PTL to prevent flooding. Non‐uniform current due to PTFE limitations. Complex design and stability challenges.	Only suitable for gaseous products. Relies on membrane quality and durability. Limited flexibility for varied setups.
CO_2_ supply	CO_2_ purging	Humidified gaseous CO_2_	
PTL on substrate	X	Needs gas diffusion layer	
Current density	<35 mA cm^−2^_geo_	>35 mA cm^−2^_geo_	
Distance between electrodes	Relatively far	Thickness of catholyte layer	Zero‐gap
CO_2_ flow rate	Low		High
Concentration of electrolyte	High conc. (e.g., 1 M KOH)		Low conc. (e.g., 0.1 M KOH)
Product phase	Gas, liquid		Gas Liquid (additional apparatus is required)

#### 2.1.1 H‐Type Cells

In electrochemical studies, traditional H‐type cells have been used to conduct the CO_2_RR [16, 25, 26] because they are easy to operate and receptive to various electrode materials and configurations. The cathode and anode chambers are filled with electrolytes and separated by an ion‐exchange membrane. A proton‐exchange membrane (PEM) is used for testing under acidic or neutral conditions, whereas an anion‐exchange membrane (AEM) is employed for experiments in basic environments. H‐type cells comprise working and reference electrodes, which are located in the cathode chamber, and a counter electrode, which is situated in the anode chamber. Because H‐type cells include a reference electrode, they are mainly used to study the attributes of catalysts incorporated onto substrates, such as intrinsic activity and reaction mechanism. Additionally, unlike in other types of electrolysis such as hydrogen evolution reaction (HER) or oxygen evolution reaction (OER), CO_2_RR systems in H‐type cells feature CO_2_, a gas dissolved in the electrolyte, as reactant. Notably, the catholyte is purged with CO_2_ gas to saturation prior to the experiments, and CO_2_ gas is bubbled throughout the reaction. The CO_2_RR in H‐type cells is significantly influenced by the solubility of CO_2_ in the catholyte. The low solubility of CO_2_ in aqueous electrolytes (33 mM under ambient conditions) [19, 27] limits the diffusion rate of CO_2_ to the cathode surface, resulting in mass‐transfer limitations and low current density [28, 29]. Additionally, owing to the physical separation between the cathode and anode, the electrolyte‐filled compartment exhibits significant ohmic losses, reducing the overall energy efficiency of the system.

**FIGURE 2:**
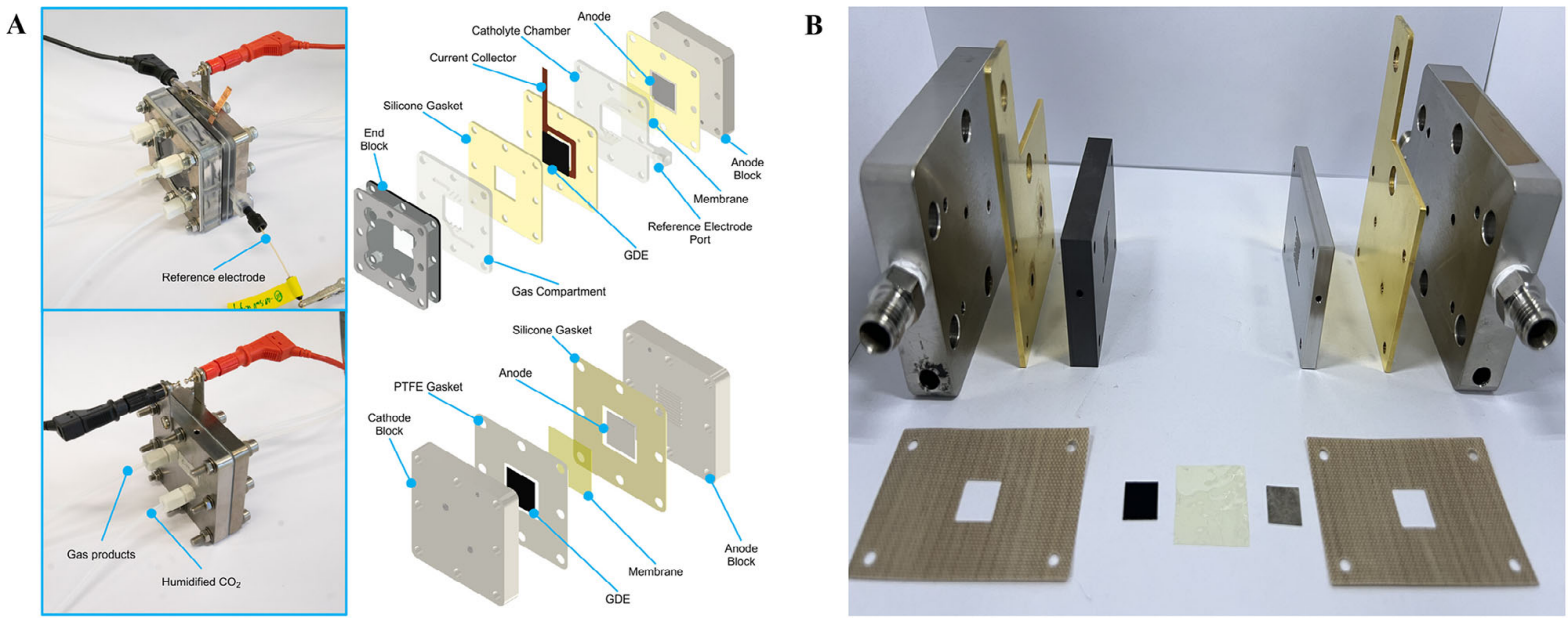
(A) Dioxide materials commercial electrolyzer: In the case of the above figure, customized parts are used for in‐situ experiments. Reproduced with permission [21]. Copyright 2023, American Chemical Society. (B) Electrolyzer with all parts customized.

Certain CO_2_RR mechanisms are sensitive to conditions such as the local pH; in these cases, the applied potential or current can vary significantly, underlining the suitability of H‐type cells for studying the reaction mechanism. Therefore, H‐type cells should be used as preliminary testing systems to evaluate the reaction rates of catalysts intended for use as cathodes in relevant industrial‐level flow‐type or MEA‐type electrolyzers and to optimize system performance. To design alternative systems for elucidating the performance of flow‐ or MEA‐type electrolyzers, two conditions must be met: (1) CO_2_ should be supplied as gas from the back of the cathode (Figure 1B), and (2) interactions should occur at the triple‐phase boundary (TPB) of the membrane, catalyst, and CO_2_ gas in the local region of the cathode.

#### 2.1.2 Flow‐Type Electrolyzers

To address the issues inherent to H‐type cells, such as the solubility of CO_2_ and ohmic loss of the electrolyte, flow‐type electrolyzers have been devised based on the MEA‐type structures traditionally employed in fuel cells and water electrolyzers [30, 31, 32]. Flow‐type electrolyzers are designed to supply reactants from the back of the electrode using a porous transfer layer (PTL) as the electrode substrate. Thus, in the CO_2_RR, CO_2_ is supplied in gas form to the back of the cathode, and a minimally thick catholyte chamber is positioned between the cathode and the membrane to minimize ohmic loss.

In H‐type cells, CO_2_ diffuses from a distance of over 50 µm to the front of the cathode; however, when CO_2_ is supplied from the back through the PTL, the diffusion path is shortened to ≈50 nm, significantly increasing mass‐transfer efficiency [12]. Separating the CO_2_ gas supply and the catholyte flow permits the use of highly alkaline electrolytes, which allows the local pH at the cathode surface to be maintained at a high level; this can suppress the competing HER and promote C–C coupling, resulting in a higher FE for C_2+_ products [33, 34]. Additionally, it can prevent the formation of salts via the reaction between CO_2_ and OH^−^‐containing compounds such as carbonates and bicarbonates. Cu‐based catalysts promote the formation of diverse gaseous and liquid products. This is advantageous because the gaseous products are separated toward the side where CO_2_ is supplied at the back of the cathode, whereas the liquid products are separated on the catholyte side. However, this separation requires the electrode to be highly hydrophobic to prevent electrolyte flooding at the back of the cathode. Generally, a carbon‐paper‐based microporous layer (MPL)—which is carbon paper coated with polytetrafluoroethylene (PTFE)‐containing carbon particles—is used to address this problem. For instance, Montfort et al. demonstrated that a PTL with superhydrophobic PTFE could significantly reduce flooding [35]. Although superhydrophobic electrodes can mitigate these problems, the nonconductive nature of PTFE leads to a nonuniform current distribution, reducing the system efficiency and stability. Therefore, structurally conductive electrodes with superhydrophobic properties must be developed.

#### 2.1.3 MEA‐Type Electrolyzers

MEA‐type electrolyzers are referred to as zero‐gap electrolyzers because they do not feature the catholyte layer [36, 37]. The cathode and anode are in complete contact with the membrane, which significantly reduces the internal ohmic resistance [38, 39]. CO_2_ gas is supplied to the catalyst through the back of the cathode PTL, while the anolyte is supplied to the catalyst through the back of the anode PTL for the OER. Thus, flow channels are worth investigating to ensure the smooth supply of reactants to the back of the electrode. The MEA‐type structure completely separates the cathode and anode with a membrane. Because the products are separated on each side, the quality of the membrane significantly influences the performance of the electrolyzer. The products must be adequately separated without crossover, and the necessary ions should be able to move appropriately; furthermore, the membrane must withstand the physical stress caused by the active flow of the reactants and products, and water should be able to move appropriately from the anode to the cathode. This design allows the system to operate at low voltages and high current densities, thereby satisfying industrial requirements. Furthermore, the compactness of MEA‐type electrolyzers, similar to that of fuel cells and water electrolyzers, can be leveraged to expand the electrode area and permit stacking, thereby enabling industrial‐scale applications. However, the structural characteristics of MEA‐type electrolyzers make them conducive to collecting only gaseous products and not liquid products. Therefore, if MEA‐type electrolyzers can be designed to produce gaseous products such as CO, CH_4_, and C_2_H_4_ with high FEs, they can become more commercially viable.

The electrolyzers are generally used by purchasing commercial products (complete CO_2_ electrolyzer, dioxide materials) (Figure 2A). In addition, some parts of electrolyzers are directly manufactured (using 3D printing) for experiments [21]. In some cases, all parts of the electrolyzer are customized and manufactured for use (Figure 2B). Usually, in such cases, factors like the corrosiveness and physical strength of each part are considered. The optimization of the structure and the selection of materials for each part are also important. Especially for the end plate or bipolar plate that constitutes the flow path (depending on the configuration of the electrolyzer being used), Ti is commonly used when an acidic electrolyte is used, and either Ti or stainless steel is used when an alkaline electrolyte is used. In addition, the material of the anode part, where the oxidation reaction occurs, is sometimes protected by plating Pt or Au on Ti. As a result, when selecting an electrolyzer, it is important to appropriately choose the structure and materials based on the experimental conditions.

### 2.2 Flow Channel

**FIGURE 3:**
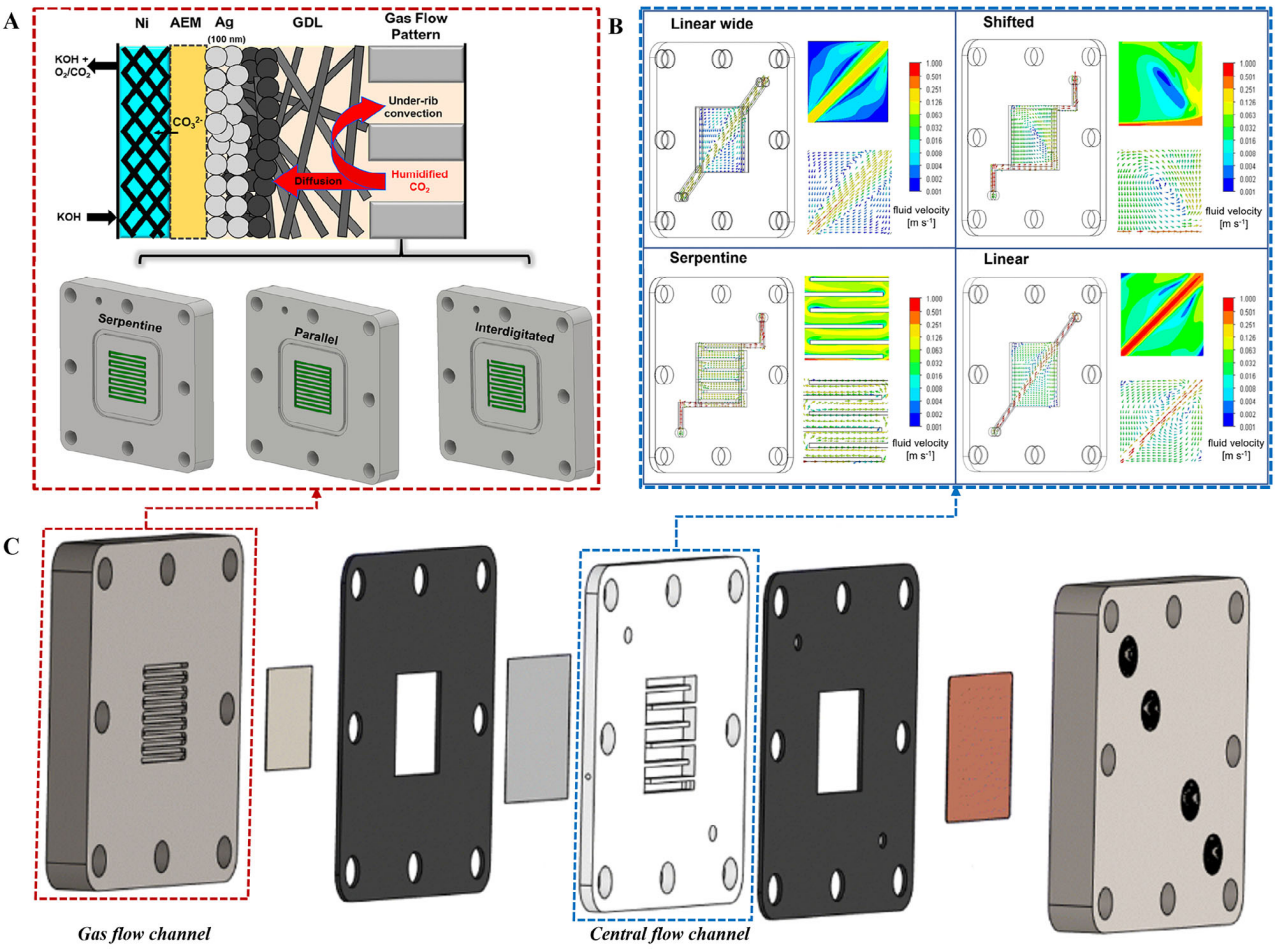
(A) Three basic configurations of the gas flow channel. Reproduced with permission [46]. Copyright 2023, American Chemical Society. (B) CFD simulation results of the studied central flow channel. (C) Schematic illustration of CO_2_RR flow‐type electrolyzers. Reproduced with permission [50]. Copyright 2023, Royal Society of Chemistry.

Each component of CO_2_RR electrolyzers must be optimized to achieve high reactivity. Advances in CO_2_RR electrode design have led to higher current densities, thereby helping boost industrial applicability [40, 41]. As the electrode area has been enhanced, the mass transport of the reactants and products has increasingly affected the current density and FE. Both MEA‐type and flow‐type electrolyzers have flow channels for supplying CO_2_ gas to the back of the cathode, with the latter also featuring an additional central channel for catholyte flow [42]. Each channel supplies reactants and emits products; therefore, a flow channel structure that enables a smooth supply of reactants and emissions of products is essential for achieving high performance. The gas flow channel directly transmits electricity to the PTE and compresses the MEA. Additionally, the flow channel in the anode compartment typically carries electrolytes such as KOH, H_2_SO_4_, and KHCO_3_. The requirements for this flow channel include physical strength to withstand the pressure of the electrolyzer, high corrosion resistance to alkalis or acids, high electrical conductivity, and nonreactivity to reactants and products. Notably, research on fuel cells and water electrolyzers has also had an impact on flow channel design [42, 43, 44]. Therefore, flow channels have typically been constructed as bipolar plates. Although various flow channel structures have been investigated, three basic configurations have generally been reported (Figure 3). The serpentine flow channel, which is the most commonly used structure, comprises a single channel arranged in a winding pattern. This single‐flow channel experiences significant pressure drops toward the outlet because of its long channel length and numerous directional changes. The parallel‐flow channel features an arrangement of multiple uniform paths to curb reactant stagnation and achieve low pressure drops. The interdigitated‐flow channel has two unconnected paths that resemble interlocking fingers. Here, the reactants pass through a PTL to move from the inlet to the outlet, inducing flow within the PTL and an increase in the area exposed to the reactants. This structure is advantageous for removing precipitates or liquids that accumulate in the PTL.

#### 2.2.1 Gas Flow Channel

As in conventional fuel cell research, the cathode component of CO_2_RR systems includes flowing gas, rendering the inlet direction and flow channel design critically important [45]. Typically, the inlet and outlet for the gas reactants are at the top and bottom, respectively. As the geometric area of the electrolyzer increases and a high SPCE is targeted, spatial variations in the reactant distribution occur along the gas flow channel of the electrolyzer as the reactants are consumed. In particular, because the area of the gas flow channel in contact with the PTL is considerably smaller than the geometric catalyst area, the gas must also move in‐plane through the PTL to reach the active catalyst sites adjacent to the current collector. If the transport from the gas flow channel to the immersed catalyst layer (CL) is not appropriately considered, certain areas of the CL may become depleted of CO_2_ even if ample CO_2_ remains in the gas flow channel. These temporal and spatial mass‐transport effects in CO_2_RR electrolyzers reduce the utility of the CL for the CO_2_RR. Subramanian et al. attempted to optimize the use of a geometric flow channel in zero‐gap CO_2_RR electrolyzers and reported methods to achieve high current density and energy efficiency [46]. Moreover, the use of Ag catalysts was analyzed by comparing the three common flow patterns (serpentine, parallel, and interdigitated) (Figure 3A). The serpentine flow channel maintained the highest CO selectivity and the lowest H_2_ selectivity, and modeling confirmed that the CO_2_ distribution within the channel was uniform. Additionally, a comparison of flooding and salt formation revealed that the flow channel should also be appropriately selected along with the PTL and membrane.

The flow channel design of bipolar fuel‐cell plates has been extensively studied [47, 48, 49]. Various factors, such as the flow channel shape, gas flow direction, flow channel cross‐section shape, channel depth, rib width, and angles within the channel, have been researched. Similarly, the most efficient flow channel design must be determined for CO_2_RR electrolyzers.

#### 2.2.2 Central Flow Channel

The central flow channel is used to facilitate the smooth separation of the CO_2_RR‐derived liquid products. To minimize the ohmic resistance, a slender 2–3 mm‐thick chamber is introduced between the cathode and the membrane to permit electrolyte flow. Generally, the flow channels for liquids are significantly influenced by gravity, and this effect is even more pronounced when both liquids and gases are transported. Therefore, liquid reactants containing gas bubbles should flow from bottom to top. Typically, the central flow channel features chambers without internal structures. Filippi et al. examined the influence of the catholyte flow compartment design on the performance and product selectivity of a CO_2_RR electrolyzer (Figure 3C) [50]. Cu‐based catalysts and high current density helped convert CO_2_ to hydrocarbons, and the primary objective was optimization of the fluid velocity distribution, bubble dynamics, and local pH conditions. Notably, four central flow channel structures were compared: linear wide, shifted, serpentine, and linear. Simulations indicated that the shifted structure demonstrated the most uniform fluid velocity distribution among the configurations. In terms of the electrolyzer performance, the shifted structure with a uniform fluid distribution exhibited the highest C_2+_ selectivity and current density among the configurations.

An important aspect of the central flow channel is the uniformity of the fluid in contact with the catalyst surface. Because the internal flow structures can reduce the active area of the catalyst, certain electrochemical reactor studies have focused on structures that do not obstruct the active area of the catalyst to control fluid uniformity and velocity [21, 51, 52]. Various structures achieved uniform mass transfer and current distribution while providing satisfactory fluid dynamics at moderate pressure drops.

### 2.3 Membrane

Membranes are classified into organic (polymer) and inorganic (ceramic and metal) variants depending on their constituent material. Inorganic membranes require complex manufacturing processes and are more expensive than polymer membranes [53, 54, 55]. Moreover, they exhibit limited ionic conductivity and selectivity [56]. Consequently, polymer membranes are preferred owing to their excellent chemical resistance, heat resistance, and electrochemical stability. Furthermore, the properties of polymer membranes can be readily adjusted by varying the materials used. Polymer membranes are suitable for electrolysis because they selectively transmit ions with different charges and low electrical resistance [57, 58]. In electrolyzers, polymer ion‐exchange membranes are used to separate the reactants of each electrode and form a closed loop of charges.

Polymer membranes comprise polymers in which the backbone has a fixed charge and the hydrophilic branches have mobile ions. The hydrophilic branches gather and form ion‐exchange channels with water during membrane fabrication. The membrane is considered an AEM when the terminal OH^−^ moiety of the branch can move through the channels, whereas it is called a cation‐exchange membrane (CEM) when H^+^ ions are mobile. Bipolar membranes (BPMs) with CEM and AEM layers have also been reported [59].

**FIGURE 4:**
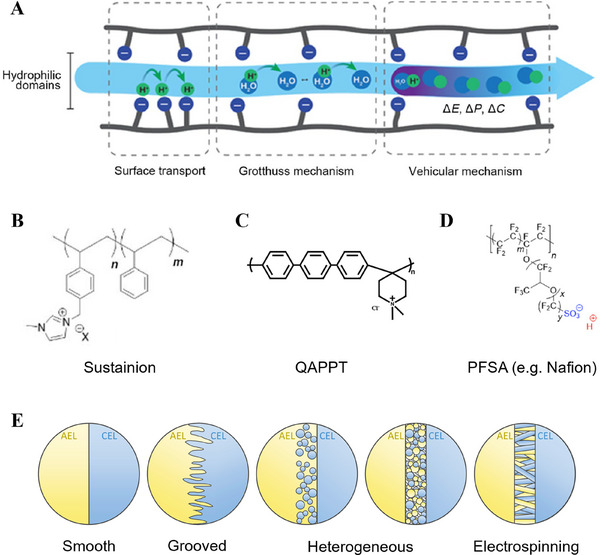
Ion transport mechanisms, polymer structures, and interfaces of bipolar membranes. (A) Schematic diagram of the ion transport mechanism in the membrane [60]. (B) Polymer structure of Sustainion [60]. (C) Quaternary ammonium poly(*N*‐methyl‐piperidine‐co‐*p*‐terphenyl) (QAPPT). Reproduced with permission [71]. Copyright 2023, American Chemical Society. (D) Perfluorinated sulfonic acid (PFSA) [60]. (E) Schematic diagrams of different interfaces of the bipolar membrane.^[81]^ Copyright 2021, Elsevier.

In the channels, ions move primarily via two mechanisms (Figure 4A) [60, 61, 62]: Grotthuss hopping, in which ions propagate through the repeated formation and separation of covalent bonds with water molecules, and vehicular transport, in which ions move owing to concentration, pressure, and potential gradients. Both mechanisms are affected by the diffusion coefficient [63], suggesting the influence of water uptake. Therefore, as much water as possible should be maintained, without compromising the ion concentration required for optimal mobility [64, 65, 66]. Additionally, the ion‐exchange capacity (IEC)—which is the amount of ions that the polymer can transport per its weight in the dry state—must be high (>1.5 meq g^−1^) to form a water channel without swelling because swelling inhibits membrane conductivity. According to Salvatore et al., water uptake should be around ≈50%–80% and swelling should be less than 15% (Table 2) [67, 68].

**TABLE 2:** Desired properties for AEMs for CO_2_RR [67].

Properties	Parameter	Required specification
Transport	Area‐specific resistance	<0.1 Ω cm^2^
	Through‐plane OH^−^ conductivity	60–100 mS cm^−1^
	Thickness	5–100 µm
	IEC	>1.5 meq g^−1^
Mechanical	Elongation at break	ε > 1.5
	Young's modulus	*Ε* ≈ 0.25 GPa
	Ultimate tensile strength	*U* ≈ 20 MPa
	Water uptake	50%–80%
	In‐plane swelling	<15%
Chemical	pH range	10–14
	Temperature	20–80°C
	Stability	Insoluble in 10 wt% alcohol
	Crossover	Minimal gas/liquid product crossover (<0.2%)
Testing conditions	Time	>1000 h
	Voltage	<3 V cell voltage
	Current density	200–2000 mA cm^−2^

#### 2.3.1 Anion‐Exchange Membranes (AEMs)

In CO_2_RR systems, the AEM transports OH^−^ and (bi)carbonate ions. Because OH^−^ is the important ion in the electrolytic reaction, the AEM must exhibit high OH^−^ conductivity. Additionally, it should be chemically stable in the pH range of 10–14 because an electrolyte with a high pH, such as KOH, is typically used. Furthermore, to be economically viable, it should exhibit long‐term stability at current densities greater than 200 mA cm^−2^ [19, 69]. Commercial membranes include Fumasep, Sustainion, and PiperION. Sustainion, a representative commercial membrane, comprises an *N*‐methylimidazolium‐functionalized styrene polymer and exhibits a conductivity of 102 mS cm^−1^ at 80°C in 1 M KOH [67]. Moreover, it operates stably at 3 V for 3800 h [70]; however, it allows unnecessary gas crossover. Another membrane, a quaternary ammonium poly(*N*‐methyl‐piperidine‐co‐*p*‐terphenyl) (QAPPT)‐based membrane, exhibits a higher conductivity of 137 mS cm^−1^ at 80°C and can thus operate with dry CO_2_ and pure water. Therefore, it has been employed in research on C_2+_ production (Figure 4B,C) [60, 67, 71].

(Bi)carbonates, which are the other ions transferred by AEMs, are formed by OH^−^ ions combining with CO_2_ and subsequently move toward the anode, causing a loss of CO_2_. This gas crossover should be minimized. Consequently, a reduction in acidic media has recently been studied. However, CO_2_ reduction occurs efficiently at a high pH; therefore, its application under basic conditions must be further studied. To achieve this, AEMs that selectively suppress the transport of (bi)carbonate ions or reduction products must be developed.

#### 2.3.2 Cation‐Exchange Membranes (CEMs)

Recent research on the CO_2_RR has largely focused on AEMs. However, the widely used commercial AEM Sustainion X37‐50 RT has chronic disadvantages. The abundant OH^−^ ions generated via the dissociation of excess water transferred from the anode promote the formation of (bi)carbonates, which combine with metal cations (Li^+^, Na^+^, and K^+^) to create salts that block the active sites of the catalyst and flow paths, hindering CO_2_ flow [72]. Additionally, as the electrolyzer dimensions increase, the CO_2_ gas supplied to the cathode can cross the anode. To solve this problem, the CO_2_RR in an acidic environment has been studied using a CEM. Unlike AEMs, CEMs transfer cations primarily by moving H^+^ ions. In CEM‐containing CO_2_RR systems, the H^+^ ions moving from anode to cathode are used directly in proton‐coupled electron transfer, which is a mechanism that suppresses the losses caused by the formation of (bi)carbonates via the reaction of OH^−^ with CO_2_, thus overcoming the problem encountered by AEMs. Moreover, according to Kim et al., the presence of H^+^ ions in the electrolyte can prevent an increase in pH by neutralizing the local OH^−^ ions generated through the cathodic reaction, thereby inhibiting (bi)carbonate formation. Furthermore, a techno‐economic analysis underscored the suitability of the CO_2_RR under acidic conditions, given the availability of inexpensive renewable electricity [73].

Typical CEMs comprise a hydrophobic perfluorinated backbone with sulfonic acid (SA) groups on its side chains [60, 74, 75, 76]. These CEMs are collectively denoted perfluorinated sulfonic acid (PFSA)‐based membranes. The terminal SO_3_^−^ moiety of the branch forms inverted micelles, creating a water channel (Figure 4D) [60], with H^+^ ions functioning as mobile units. The presence of numerous SO_3_^−^ groups implies a high degree of sulfonation, with a low degree of sulfonation suggesting reduced conductivity. However, an excessively high value causes durability issues [77]. Nafion is extensively used as a CEM in CO_2_RR devices. Different versions of Nafion membranes, such as Nafion 212, Nafion 115, and Nafion 117, can be selected depending on the characteristics required for the experiments, such as water uptake, conductivity, and thickness. However, they undergo excessive swelling in the presence of high concentrations of alcohols when integrated into direct methanol fuel cells and are under increased scrutiny owing to toxicity concerns [78]. This issue is worth considering for CO_2_RR systems because alcohols are considered products. Alternatively, other commercial PFSA‐based CEMs such as Aquivion and Fumapem can be used. Furthermore, CEMs with remarkably high IECs can often induce excessive H^+^‐ion transfer, promoting competing reactions such as the HER in CO_2_RR systems, thereby underlining the importance of selecting a CEM with suitable properties.

#### 2.3.3 Bipolar Membranes (BPMs)

BPMs are a combined form of the AEM and CEM. Cations and anions move through each layer, forming or decomposing water between the two layers. The former one where the CEM on the anode side is called forward‐bias mode, and the latter one CEM on the cathode side is called the reverse‐bias mode. Fumasep BPM is the only commercial BPM.

In the forward‐bias mode, anions and cations meet to form a product at the AEM–CEM interface, with water or CO_2_ formed during CO_2_ reduction. Removing the product is vital to prevent layer separation and increase cell voltage [79], indicating the necessity of a highly porous environment. For instance, Xu et al. effectively removed CO_2_ by introducing a microchannel solid electrolyte between the membranes [80].

In the reverse‐bias mode, a large interfacial area is required to ensure mechanical durability against local water dissociation. Attempts have been made to maximize the surface area of the interface by roughening one membrane surface, stacking other layers, and tying gaps. Moreover, a heterogeneous interface has been created by completely surrounding the resin particles of the ion‐exchange polymer with a matrix or polymer particles of a second layer. Furthermore, a method has been established to randomly distribute nanofibers at the two interfaces by electrospinning to prevent layer separation (Figure 4E) [81].

Alternatively, BPMs can be fabricated by simply overlapping two commercial membranes. For instance, Disch et al. created pores on an AEM film and then carefully overlapped it with a CEM [82]. Xie et al. introduced a TiO_2_ layer between a CEM and AEM as a water‐dissociation catalyst to fabricate a sandwich‐structured BPM [83]. Wu et al. inserted an SnO_2_ layer between a CEM and an AEM as a water dissociation catalyst; however, the membranes were fabricated by spraying [84].

### 2.4 Electrolyte

Various electrolytes have been employed in CO_2_RR systems, with most studies having been conducted using aqueous solutions. Water is inexpensive, abundant, and can be easily employed in laboratory‐scale research. Alkali‐metal‐based electrolytes (such as KOH and KCl) are commonly used as additives to prepare aqueous ionic liquids. Notably, metal cations play an important role in the CO_2_RR. However, the HER occurring with the water during electrolysis competes with the CO_2_RR. Moreover, aqueous electrolytes exhibit limited CO_2_ solubility and CO_2_ mass transfer. Consequently, organic solvents and additives have been targeted to overcome these challenges. However, these alternatives face challenges owing to their high price and toxicity.

#### 2.4.1 Inorganic Electrolytes: Aqueous Ionic Liquids and Cation/Anion Effects

Potassium‐based electrolytes have been extensively used for the CO_2_RR, with appropriate anions selected depending on the pH conditions of the electrolyzer. An aqueous KOH solution and KHCO_3_ have been used as the electrolyte under basic and near‐neutral conditions, respectively, whereas KCl and K_2_SO_4_ have been used in neutral environments. For the CO_2_RR under acidic conditions, the pH is adjusted by adding acids (such as H_2_SO_4_) to a neutral electrolyte. The electrolytic reaction is significantly affected by the ions generated on the electrodes and the ions transferred through the membrane. Because alkali metal cations also play a major role in the CO_2_RR, it is also a key factor to select an appropriate cation.

**FIGURE 5:**
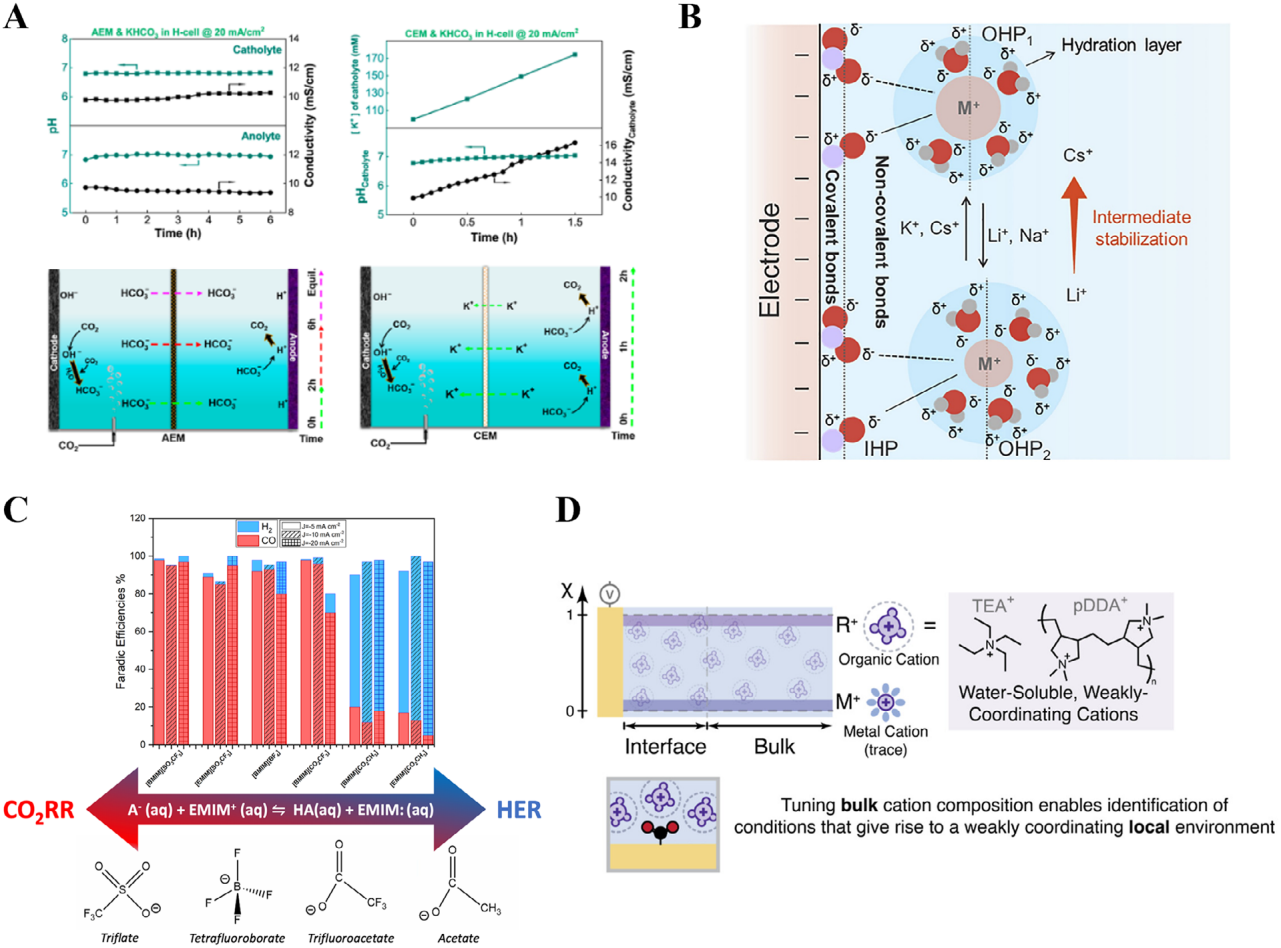
Research about different electrolytes in CO_2_RR. (A) pH value of catholyte and anolyte to reaction time on both CEM and AEM, diagrams about CO_2_RR in aqueous KHCO_3_. Reproduced with permission.^[85]^ Copyright 2024, American Chemical Society. (B) Schematic diagram showing cation effects with alkali metal electrolyte.^[91]^ Copyright 2022, Elsevier. (C) FE values of the gaseous products generated during the CO_2_RR with different organic cations and anions.^[96]^ (D) Schematic diagram showing replacement of metal cations with bulk organic cations and their weakly coordinated local environment. Reproduced with permission.^[97]^ Copyright 2023, American Chemical Society.

In AEM‐based CO_2_RR systems, the anions in the electrolyte are transferred at the beginning of the electrolytic reaction. However, as the reaction proceeds, the (bi)carbonate formed by the combination of the OH^−^ ions produced at the cathode with CO_2_ becomes the major charge carrier. Consequently, the pH of the catholyte and anolyte gradually increases and decreases, respectively. The transferred (bi)carbonate interacts with the H^+^ ions formed at the anode to form CO_2_, causing CO_2_ gas loss. When KCl is used, ClO^−^ ions are generated on the anode side and induce the degradation of the AEM. KHCO_3_ is suitable to prevent these changes (Figure 5A) [85].

In the early stages of CEM‐based electrolysis, the motion of dissolved metal cations is the primary mechanism. However, as the reaction proceeds, the H^+^ ions formed at the anode gradually become the main charge carriers. When KCl is used, Cl_2_ gas is formed at the anode side; consequently, the number of ions in the anolyte gradually decreases, resulting in poor conductivity, which causes an increase in cell voltage. When KHCO_3_ is used as the electrolyte, the H^+^ ions formed at the anode also convert (bi)carbonate to CO_2_, leading to a decrease in conductivity [85].

The effects of cations in the CO_2_RR have also been studied. For instance, the electric field at the solid–liquid interface is affected by the type and diameter of hydrated ions. Moreover, it is involved in the intermediate stage of the electrolytic reaction and affects the selectivity for the final product [86]. Evidently, the hydrated radius decreases and the CO_2_ reduction activity increases in the order of Li, Na, K, and Cs (Figure 5B) [87, 88, 89, 90, 91].

#### 2.4.2 Organic Electrolytes: Nonaqueous Systems, Organic Additives, and Solid‐State Polymers

Organic solvents such as acetonitrile (ACN) and dimethyl formamide (DMF) generally exhibit higher CO_2_ solubility and contain fewer protons than those of aqueous electrolytes. These attributes have been targeted in attempts to improve the overall efficiency of the CO_2_RR using organic solvents [28]. However, organic solvents cannot prevent decomposition during electrolysis. Using gas chromatography, Kim et al. detected overestimated signals of CH_4_ when Cu catalysts reacted in commonly used organic solvents such as ACN, DMF, dimethyl sulfoxide (DMSO), propylene carbonate (PC), and 3‐methoxypropionitrile (MPN). Significant amounts were detected in the case of ACN, but amounts were smaller in the case of DMF [92].

The effects of organic anions and cations have also been investigated. The most common organic electrolytes used for the CO_2_RR include imidazolium‐based cations such as 1‐butyl‐3‐methylimidazolium [BMIM]^+^ and 1‐ethyl‐3‐methylimidazolium [EMIM]^+^ because of their high CO_2_ capture ability, with imidazolium‐halide‐based organic ionic liquids (such as those containing [BF_4_]^−^ and [PF_6_]^−^) drawing considerable interest owing to the stability of the cation ring and the hygroscopicity of the anion [93]. However, the use of fluorine‐based anions is under scrutiny owing to their potential decomposition into toxic HF [94, 95]. In research conducted to overcome this limitation, Fortunati et al. found that acetate anions (strong Lewis bases) enhance CO_2_ capture and H_2_ evolution (FE_CO _< 20%), whereas fluorinated anions (weaker Lewis bases) favor CO_2_ electroreduction (FE_CO_ upto 97%) (Figure 5C) [96]. Other organic cations have also been explored. For example, Weng et al. recently replaced metal cations in aqueous electrolytes with organic materials and found that Ag‐based catalysts exhibited higher current densities and FEs than those of certain metal cations (Figure 5D) [97].

Additionally, the introduction of an all‐solid‐state polymer electrolyte into BPMs has been studied. Styrene‐divinylbenzene copolymer microspheres with SA functional groups were used to form a layer between the AEM and CEM, and the liquid that accumulated in the porous solid layer was ejected with nitrogen to obtain high‐purity formic acid [98]. Solid‐state polymer electrolytes have also been explored in other studies [99, 100].

### 2.5 Inlet CO_2_

#### 2.5.1 Gaseous CO_2_

**FIGURE 6:**
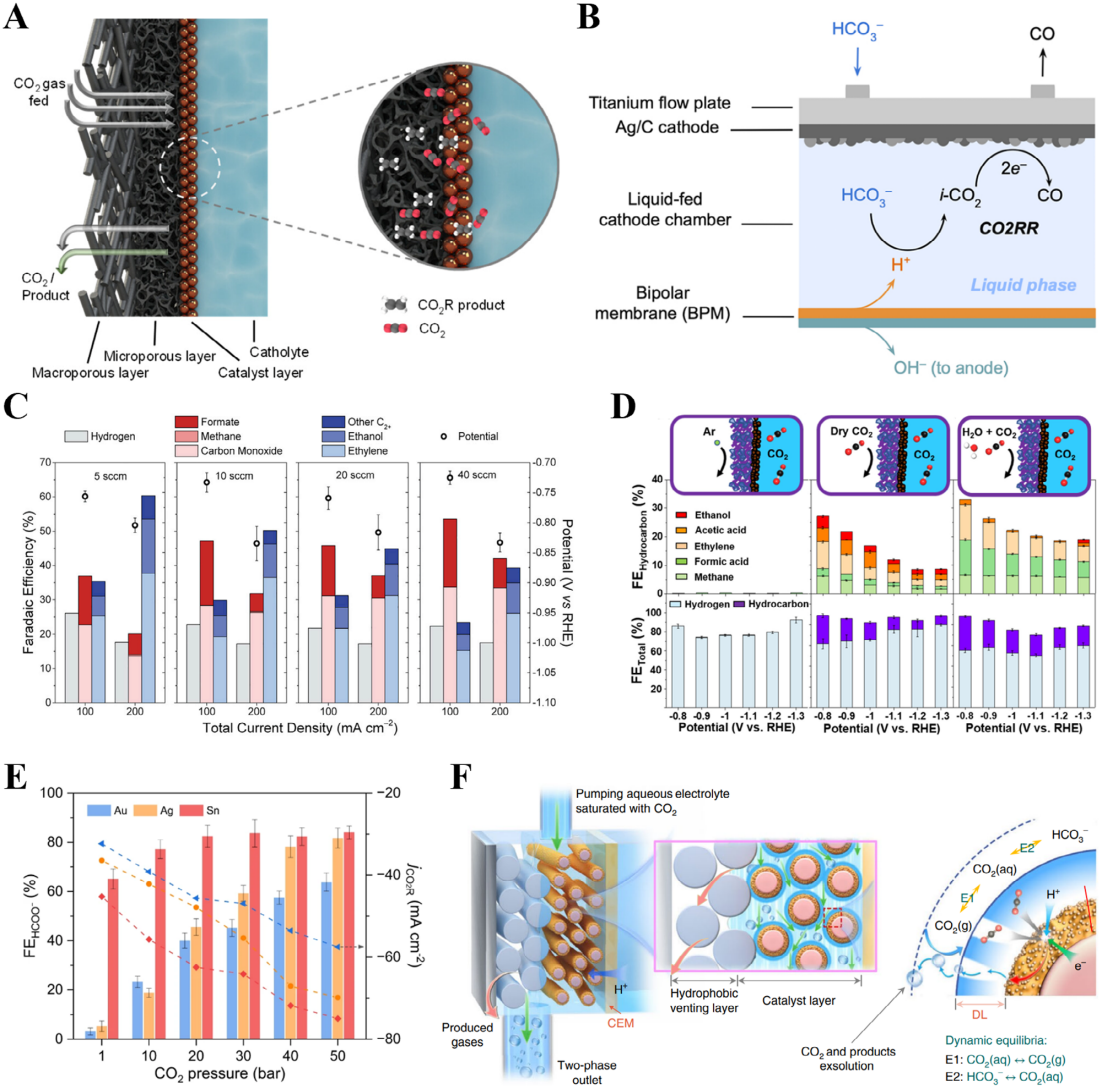
(A) Schematic of an electrochemical gaseous CO_2_RR PTE cathode and its fundamental components. Reproduced with permission [12]. Copyright 2024, American Chemical Society. (B) Schematic of CO_2_ conversion in the cathode compartment of a liquid‐fed bicarbonate electrolyzer. Reproduced with permission [124]. Copyright 2023, American Chemical Society. (C) Faradaic efficiencies of the major products and the applied potential at various CO_2_ feed flow rates at different current densities. Reproduced with permission [113]. Copyright 2020, Elsevier. (D) Illustration and FE of the three gas conditions: Ar (0% CO_2_), dry CO_2_, and humidified CO_2_, explored in the gas chamber of the hybrid reactor. Reproduced with permission [114]. Copyright 2022, American Chemical Society. (E) FEs toward formate and CO_2_RR partial current densities on Au, Ag, and Sn catalysts under different pressures at −1.1 V versus RHE. Reproduced with permission [128]. Copyright 2022, Springer Nature. (F) Illustration of the FTDT cell for pumping CO_2_‐saturated catholyte throughout a porous electrode with CO_2_ exsolution from the dynamic equilibria E1 and E2. Reproduced with permission [129]. Copyright 2022, Springer Nature. CEM, cation exchange membrane; GDL, gas diffusion layer.

H‐type cells have traditionally been used as aqueous‐fed CO_2_ systems, allowing the direct use of captured CO_2_ [19]. However, these systems face challenges owing to the low solubility and diffusion rate of CO_2_ [28, 101, 102, 103], making it difficult to achieve a high current density. Gaseous CO_2_ systems have been developed to address these issues [104]. In these systems, CO_2_ penetrates the back of the porous electrode and reacts with the catalyst, following which the products are expelled (Figure 6A) [12, 105, 106]. These systems offer advantages such as high CO_2_ concentrations and short diffusion paths, enabling efficient reactions [107]. The input CO_2_ flow rate and humidity are crucial operational parameters for modulating the selectivity and energy efficiency.

The CO_2_ flow rate significantly affects the performance metrics of electrochemical reactors, particularly the FE and overall reaction rate [21, 37, 108]. A high flow rate enhances mass transfer, ensuring sufficient supply to the reaction sites, which is crucial for maintaining a high current density and efficiency [109]. However, an excessively high rate can reduce the residence time within the reactor, limiting the interactions with the catalyst and thereby lowering efficiency [110, 111]. Additionally, it can induce hydrodynamic instability, thereby increasing mass transfer resistance and impairing catalyst performance. Conversely, a low flow rate can create depletion zones near the electrode surface, limiting transfer and reducing the current density and overall efficiency [112]. The optimal rate balances these factors, ensuring an adequate supply and sufficient interactions with the catalyst. Evidently, an intermediate flow rate maximizes the efficiency of generating specific reduction products, as it optimally aligns the supply with the consumption rate at the catalyst surface (Figure 6C) [113]. Therefore, fine‐tuning the flow rate is essential for optimizing reactor performance and balancing the mass transfer and reaction rates, thus achieving the highest possible efficiency and product selectivity.

Humidification of the CO_2_ feed during reduction reactions is crucial for optimizing the reaction environment and enhancing the efficiency of the electrolyzer (Figure 6D) [114]. An effective method for achieving this is to bubble the gas through a water bath, allowing it to absorb water vapor and become humidified. This process provides the protons necessary for the reaction, thereby increasing the hydrocarbon production efficiency. Furthermore, it suppresses the HER, thus improving the CO_2_ reduction efficiency in an MEA configuration [115]. Maintaining the catalyst stability and performance is another benefit, as adequate hydration prevents salt precipitation and electrolyte flooding [116]. A humidified CO_2_ feed improves reactant diffusion, leading to higher local concentrations at the catalyst surface, thus enhancing the reaction rate and selectivity. Appropriate electrode hydration ensures high ionic conductivity and efficient ion transport between the anode and cathode [115]. These measures help avoid issues such as inactive catalytic sites and poor ionic conduction under dry conditions.

#### 2.5.2 Aqueous CO_2_

Gas‐phase CO_2_ reduction systems, including both flow cell and MEA configurations, have been employed to achieve selective CO_2_ conversion at high current densities. However, these gas‐phase systems frequently utilize alkaline electrolytes or AEMs to achieve high selectivity. Consequently, significant issues arise, including carbonate formation with alkaline electrolytes or CO_2_ crossover with AEMs [117, 118]. These problems necessitate the supply of additional energy for the electrolyte or CO_2_ recycling. Furthermore, because the concentration of CO_2_ in industrial flue gases is too low for efficient reactions, the CO_2_ streams must be enriched and purified, which is an expensive process [14, 119].

Recently, systems with carbonate and bicarbonate feeds have been developed to integrate CO_2_ capture and conversion into a single process [119, 120, 121, 122]. By reacting protons derived from a BPM with (bi)carbonate, CO_2_ is produced in situ within the reactor (Figure 6B) [19, 124]. This approach eliminates the energy‐intensive step of extracting CO_2_ from capture solutions, thus enhancing carbon utilization [123]. Additionally, gaseous products can be produced at high concentrations because they do not mix with the CO_2_ feedstock [123], enabling efficient CO_2_ reduction without the need for cost‐prohibitive oxygen removal steps, which is required for gaseous CO_2_ owing to the thermodynamic favorability of oxygen reduction [124]. Despite these benefits, these systems must overcome challenges such as low current densities and preference for the HER owing to the naturally low solubility of CO_2_ [125]. Therefore, methods to convert dissolved CO_2_ into gaseous CO_2_ must be established, and the development of electrodes optimized for these conditions—which differ from those used in gas‐fed systems—should be prioritized.

High pressures have been used to enhance the solubility of CO_2_ in aqueous solutions under ambient conditions [126]. Notably, the concentration of CO_2_ increases from 0.03 M at ambient pressure to 1.16 M at 50 bar [127]. Huang et al. found that various catalysts (Cu, Au, Ag, and Sn) exhibited increased selectivity toward formate at a pressure of 50 bar (Figure 6E) [128] owing to the high CO_2_ adsorption on the catalyst surface and low proton concentration, which promoted the production of formate. Wen et al. designed an electrolyzer that utilized the forced convection of an aqueous CO_2_‐saturated catholyte through a porous electrode (Figure 6F) [129]. The CO_2_ supply was accelerated by the increased exsolution of gaseous CO_2_ from dissolved CO_2_ and bicarbonate owing to the decrease in local pressure, which shortened the CO_2_ diffusion distance and increased the concentration of CO_2_ near the catalyst. The system achieved a maximum current density of 3.37 A cm^−2^ with an Ag‐based catalyst.

### 2.6 Porous Transport Electrodes

PTEs are porous hydrophobic electrodes that significantly improve the CO_2_ mass transport to the catalyst by truncating the diffusion pathway [32, 130, 131]. They are designed to efficiently facilitate the transfer of gases and liquids to the catalyst surface while maintaining electrical conductivity and mechanical stability [32]. PTEs comprise two primary layers: the PTL and CL. The PTL supports gas distribution and water management, thereby significantly affecting the overall PTE performance. The CL, which is applied on top of the PTL, facilitates the electrochemical reactions necessary for CO_2_ reduction by reducing the overpotential and enhancing selectivity toward the desired product. Each layer plays a specific role in the overall PTE performance in CO_2_ reduction.

#### 2.6.1 Porous Transport Layer

The primary functions of the PTL include facilitating an efficient gas distribution, managing water within the electrode, and providing structural support to the CL. To achieve optimal performance, PTLs must exhibit high electrical conductivity, sufficient porosity for porous transport, and hydrophobic properties to prevent flooding and thereby ensure that active sites and gas pathways remain unimpeded [132]. Typically, PTLs are fabricated from carbon‐based materials and have either single‐ or dual‐layer structures. Carbon materials offer many advantages, such as natural abundance, customizable porous structure, high surface area, good electrical conductivity, high‐temperature stability, environmental friendliness, and affordability [133]. Single‐layer PTLs are made from carbon materials mixed with hydrophobic binders such as PTFE, whereas multilayer PTLs include an additional MPL to further facilitate water management and porous transport [133].

Single‐layer PTLs are typically macroporous substrates fabricated from carbon materials. Commercial nonwoven carbon PTLs, such as those from Toray, Freudenberg, Avcarb, and Sigracet, are available in various thicknesses and PTFE contents and have been used extensively in numerous electrochemical studies [134]. However, single‐layer PTLs have drawbacks such as susceptibility to flooding, limited durability, and mechanical instability. Flooding occurs when the electrolyte blocks gas pathways, thus reducing the electrochemical reaction efficiency [135, 136]. The absence of an MPL in single‐layer PTLs renders water management challenging and leads to performance instability. Additionally, the hydrophobicity of these PTLs degrades over time, exacerbating flooding and leading to microcracks and structural weaknesses that compromise their mechanical integrity during long‐term operation [105]. Consequently, single‐layer PTLs are increasingly being replaced by more advanced multilayer designs.

Multilayer PTLs have been developed to mitigate flooding in fuel cells [137, 138]. These PTLs consist of an MPL applied over a macroporous carbon layer [139]. The MPL is a thin layer with small pores and typically comprises carbon black powder mixed with hydrophobic agents such as PTFE [140, 141, 142]. This composite structure exhibits hydrophobicity superior to that of the single‐layer designs, effectively preventing flooding by maintaining a balance between gas and liquid phases at the electrode interface [143]. The incorporation of an MPL not only prevents flooding but also increases the surface area and improves the interfacial electrical connections, thereby enhancing the overall electrode performance.

**FIGURE 7:**
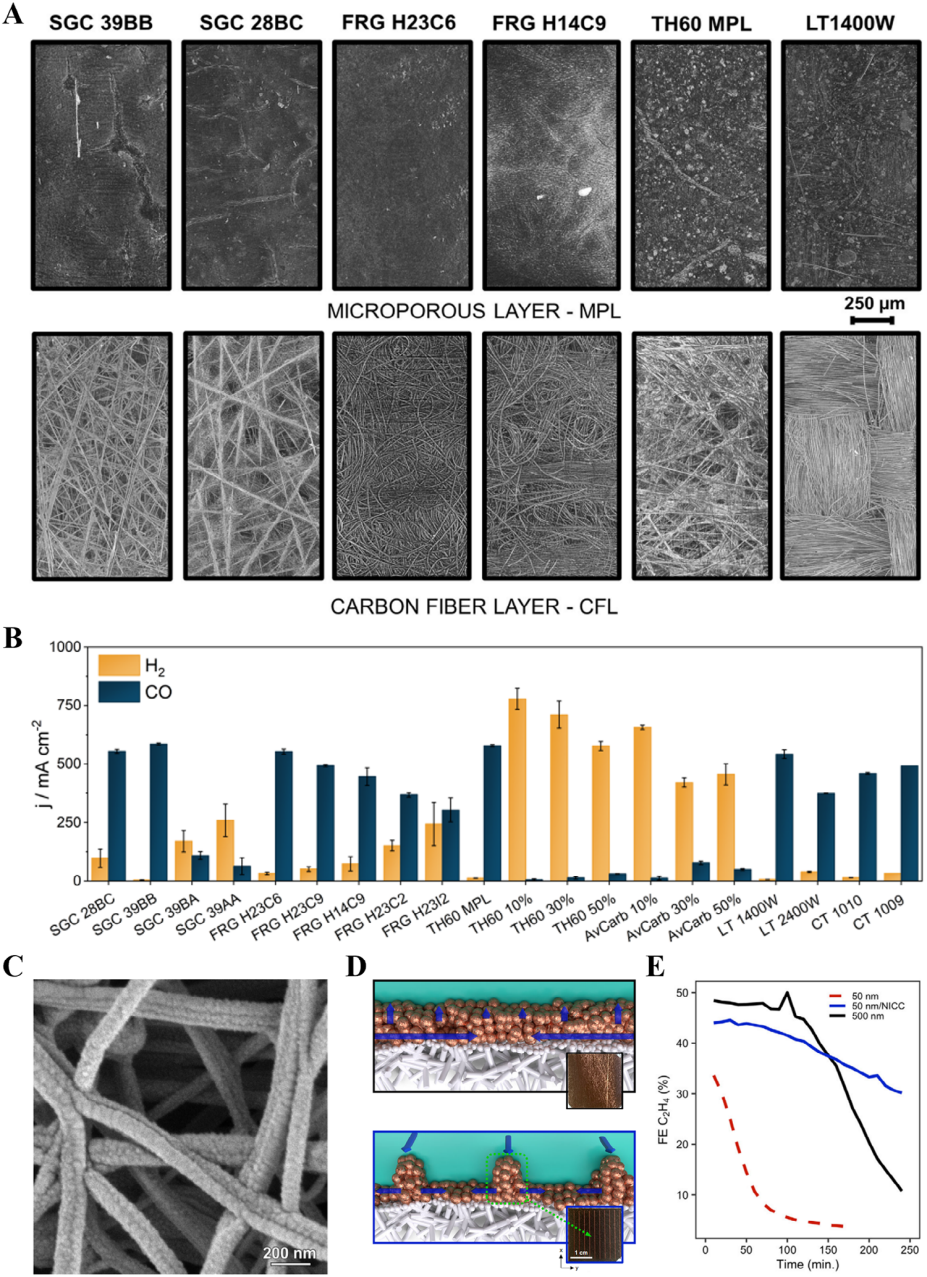
(A) Structure of different commercially available carbon gas diffusion layers observed through X‐ray computed tomography. (B) Comparing commercially available carbon PTL‐based electrodes in the CO_2_ reduction reaction at MEA with Ag catalyst. Reproduced with permission [144]. Copyright 2023, Springer Nature. (C) SEM image of Cu nanoparticles sputtered on the PTFE PTL. Reproduced with permission [196]. Copyright 2021, Springer Nature. (D) Sketches of a traditional ePTFE/Cu (top) and ePTFE/NICC/ Cu electrode (bottom). Current collection lines are depicted in blue. (E) Observed selectivity toward ethylene for the three designs at a constant potential of ≈–0.55 V versus RHE. Reproduced with permission [35]. Copyright 2023, Springer Nature.

Samu et al. systematically evaluated the performance of 20 commercially available PTLs in the CO_2_RR using an MEA [144]. In Figure 7A, the structure of various commercially available carbon‐based PTLs has been systematically characterized using X‐ray computed tomography, revealing distinct differences in porosity, fiber orientation, and MPL coverage. Furthermore, these structural variations, such as the presence or absence of an MPL, the degree of PTFE loading, and the anisotropy in carbon fiber alignment, can significantly influence CO_2_ electroreduction performance, as demonstrated through quantitative correlations between GDL architecture and catalytic activity, selectivity, and mass transport characteristics (Figure 7B). The PTLs with an MPL exhibited significantly higher CO_2_RR selectivity than that of the MPL‐free variants, achieving over 90% FE for CO production. Moreover, thicker PTLs demonstrated more stable performance during long‐term electrolysis, and the presence of more cracks facilitated gas and water management [145, 146].

Consequently, catalysts that favor gaseous products are used in MEA systems, which do not require liquid–gas separation [147]. Moreover, controlling flooding is essential for the long‐term performance of these systems [148], with the use of a thick MPL containing numerous cracks being beneficial for water management [145]. However, in flow cells—which are commonly used to generate liquid products—cracks can have detrimental effects. The use of MPLs with fewer cracks can enhance C_2+_ production during high‐current flow cell operations [149]. Additionally, even if there is a risk of flooding, the use of a thick woven carbon cloth can help maintain a stable operation by allowing the infiltrating electrolyte to fill the large voids between the fiber bundles and then drain before occupying the smaller pores [150].

However, obstacles to long‐term operation exist even in the use of hydrophobic MPLs [136, 151]. The hydrophobicity of the carbon PTL of the PTE decreases as current flows through the PTL; this phenomenon and the precipitation of hygroscopic carbonate salts intensify electrolyte flooding into the PTE pore structure [152, 153]. An effective approach to increasing PTE hydrophobicity is to use a superhydrophobic PTFE membrane as the PTL (Figure 7C), that is, a polymeric support material formed by coarse laminates of polyethylene or polypropylene [150, 154, 155]. While this approach mitigates flooding, problems with current distribution and stability persist owing to the nonconductive nature of PTFE.

Using infrared thermography, van Montfort et al. demonstrated that the incorporation of a thin CL (≈50 nm) on the PTFE PTL led to significant current density disparities, hindering performance and stability (Figure 7D,E) [35]. Noninvasive current collectors comprising 1‐µm‐thick copper busbars significantly improved the current distribution and electrode stability, achieving an FE over 30% for C_2_H_4_ production even with a 10‐fold thinner CL.

#### 2.6.2 Catalyst Layer

The CL is the surface of an electrode where catalytic materials are deposited to enable the adsorption and activation of reactants and transfer of electrons, thereby facilitating efficient reduction reactions [22]. Various transition and noble metals have been used to catalyze the CO_2_RR, with specific metals selected based on the desired reaction product. For example, Ag, Au, and Zn are primarily selective in producing CO [156, 157, 158], whereas Sn, In, Pb, and Bi predominantly yield HCOOH [159, 160, 161, 162, 163]. Notably, Cu is the sole material capable of producing hydrocarbons and alcohols such as C_2_H_4_ and C_2_H_5_OH [164]. These catalytic metals, each possessing unique properties and reaction selectivities, can generate various products through different CO_2_RR pathways [165, 166, 167].

CO_2_RR catalysts have been prepared in various forms including nanoparticles [168, 169, 170], single‐atom/metal–nitrogen–carbon (M–N–C) structures [171, 172, 173, 174, 175, 176], core–shell configurations [177, 178], and metal–organic frameworks (MOFs) [179, 180], each suited for specific reactions. Nanoparticles, which are synthesized using colloidal methods, have large surface areas [181]. Single‐atom/M–N–C structures, which are constructed by dispersing metal atoms in nitrogen‐doped carbon via pyrolysis, provide high selectivity and stability [182]. Core–shell configurations, which are produced through sequential deposition and feature a core metal coated with a different metal shell, optimize the selectivity [178, 183, 184]. MOFs, which are synthesized by integrating metal ions with organic linkers, offer high surface areas and tunable properties [185]. These catalyst forms are effective for the CO_2_RR because of specific advantages.

For particulate catalysts, the use of ink for catalyst deposition is an ideal method for achieving a uniform, controlled coating. This method allows scalability and the use of specific catalyst material compositions [186]. The traditional method of applying catalysts to electrodes has several advantages, including homogeneous distribution of the catalyst, precise control over the layer thickness and loading, scalability for mass production, compatibility with various materials, customization of the formulation, and cost‐effectiveness [187]. However, this method has some disadvantages, such as the complexity of the ink formulation, the need for post‐deposition drying and sintering, potential aggregation of catalyst particles, the possibility of binders blocking active sites, and solvent compatibility issues [188, 189].

Unlike conventional electrocatalysts fabricated using ink, self‐supported electrocatalysts are working electrodes in which the catalysts are grown directly on specific conductive substrates, eliminating the need for additional binders or supports [190]. This method offers several advantages, including enhanced electron transfer owing to direct growth on conductive substrates [191, 192], exposure of more active sites without using binders [193], and improved mechanical stability by preventing the detachment of active materials [194]. However, these catalysts also face challenges such as the complexity and cost of synthesis methods, difficulties in achieving uniform catalyst distribution, and scaling up production for commercial applications [195]. Nevertheless, self‐supported catalysts often exhibit superior performance in the CO_2_RR, making them promising systems for research and development [196].

##### 2.6.2.1 Ink‐Based CLs

The CL is typically formed using catalyst ink that contains catalyst powders, a solvent (such as water, ethanol, and isopropyl alcohol), and a nonvolatile ionic binder (or ionomer) that acts as a conductive scaffold (for example, Nafion, and XB‐9) [189, 197, 198, 199]. Carbon black is commonly used as a catalyst support, loaded with nanocatalysts, or mixed with catalyst ink to enhance the electrical conductivity of the CL. When the catalyst ink is applied to the PTL, the solvent evaporates, forming a microstructure [113]. Various factors influence the overall performance of PTEs, including the catalyst ink composition (ionomer‐to‐catalyst ratio), choice of solvent, evaporation rate, conditions, and ink deposition method.

**FIGURE 8:**
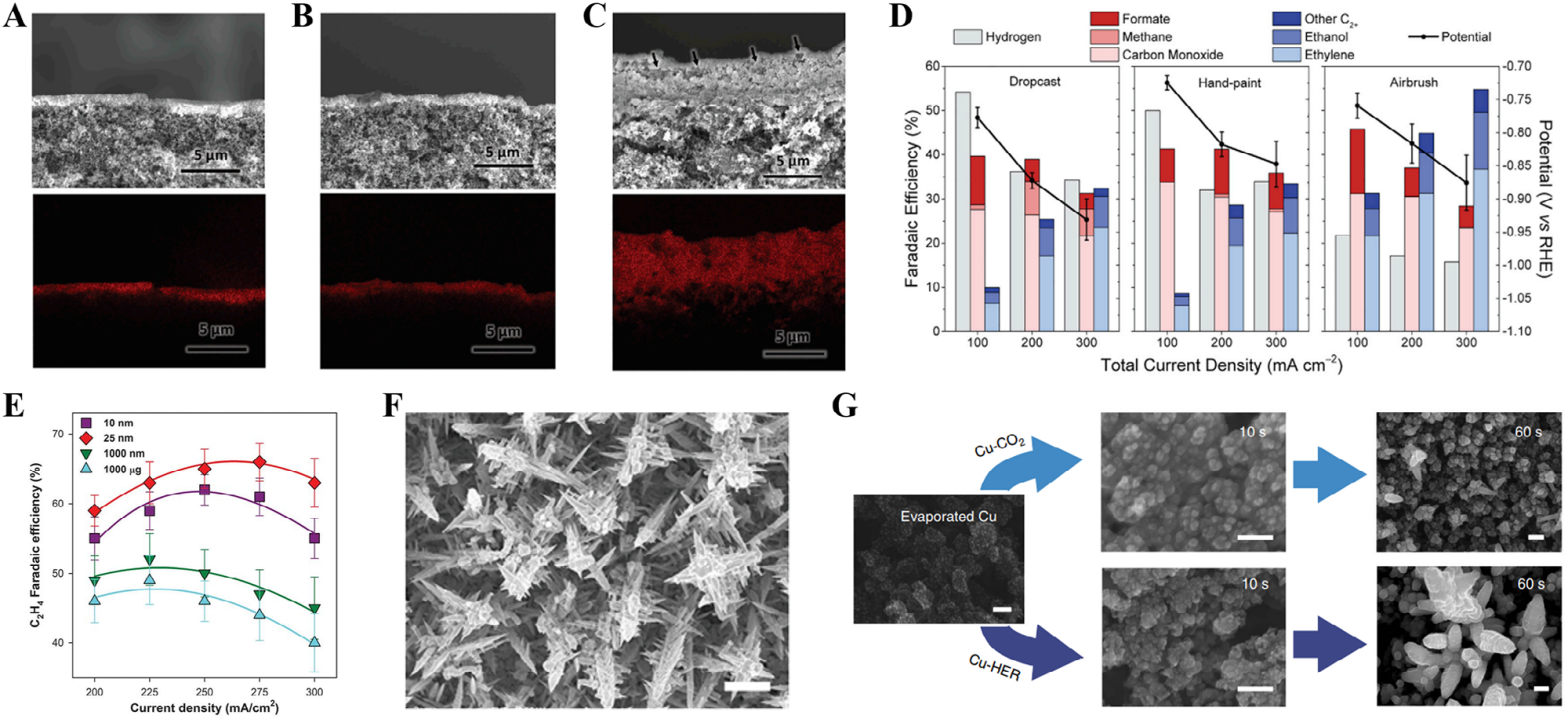
(A–C) Cross‐sectional SEM images and their energy‐dispersive X‐ray spectroscopy (EDS) elemental mappings of Cu for catalyst layers prepared by dropcasting (A), hand‐painting (B), and airbrushing (C). (D) FEs of the major products and the applied potential at different current densities for various ink‐based catalyst deposition methods. Reproduced with permission [113]. Copyright 2020, Elsevier. (E) C_2_H_4_ FEs in the current density range of 200 to 300 mA cm^−2^, showing the increased C_2_H_4_ selectivity of the abrupt reaction interface samples (10 and 25 nm) compared with that of thicker samples (1000 nm and 1000 mg) that allow for a more distributed reaction. Reproduced with permission [154]. Copyright 2020, American Association for the Advancement of Science. (F) SEM image of hierarchical Cu dendrites. Reproduced with permission [215]. Copyright 2021, American Chemical Society. (G) The time‐dependent morphological change of Cu‐CO_2_ (upper arrows) and Cu‐HER (lower arrows) during electrodepositions at 400 mA cm^−2^ in 1 M KOH containing the copper precursor. Reproduced with permission [216]. Copyright 2019, Springer Nature.

Typical ink‐based deposition methods include air brushing, drop casting, and hand painting [200]. Drop casting and hand painting can result in uneven CLs with agglomerated catalyst particles, leading to fewer electrochemically active sites for reactions than those obtained by air brushing, which tends to produce a more uniform CL (Figure 8A–D) [113].

The ionomer content also significantly affects the PTE performance by modifying the CL microstructure, porous transport, and catalytic pathways; moreover, the ionomer can even function as a co‐catalyst in the reaction [189, 199]. The ionomer forms an interconnected network that creates pores within the CL, links the catalyst particles, and conducts protons/ions, which is crucial for proton transfer. An inadequate ionomer content leads to poor ion transport within the CL, aggregation of the catalyst powder, and high ohmic resistance. Conversely, an excessive ionomer content can reduce the contact of the CL with the electrolyte, lower the open pore volume, reduce gas permeability, and increase mass transport polarization. Notably, AEM‐based ionomers and PTFE are garnering attention as alternatives to Nafion [201, 202, 203, 204].

##### 2.6.2.2 Self‐Supported CLs

Ion‐binder‐free self‐supporting CLs can be fabricated by electrodeposition, sputtering, pulsed laser deposition, and ion beam deposition [205]. Among these techniques, sputtering is notable for its cost‐effectiveness and simplicity in achieving uniform CLs as thin as 10 nm on both conductive and nonconductive PTLs [206]. This method ensures high stability and strong adhesion to commercial PTLs, with precise control over the layer thickness realized by adjusting the sputtering duration or number of cycles [207]. Additionally, it facilitates research on bimetallic materials because of its ability to achieve an exact compositional ratio [208]. Sputtering is particularly beneficial for PTFE‐based PTLs in contrast to traditional ink‐based techniques, which are impractical owing to the hydrophobic nature of the material.

Dinh et al. demonstrated the fabrication of Cu‐based PTE using sputtering to deposit catalyst layers of varying thicknesses on PTFE PTL (Figure 8E) [154]. Their findings revealed that 25 nm provided enough active sites in the optimal catalyst layer for C_2_H_4_ FE peaking at 66% at 10 M KOH. However, thicker layers beyond 25 nm produced excessive hydrogen evolution, hindering the desired C−C coupling by being largely devoid of CO_2_. Although thinner layers might have fewer active sites, they exhibit higher FE at elevated current density, balancing out the conversion rate.

Electrodeposition is another effective ink‐free method for uniformly depositing pure metals or alloy catalysts on conductive substrates, rendering it suitable for large‐scale PTE production [209, 210]. During electrodeposition, metal ions are reduced on the cathodic substrate, forming a film that adheres directly, without the need for an ionomer [211, 212]. The morphology and electrocatalytic performance of the resulting films depend on factors such as the composition of the electroplating solution, additives, substrate type, applied potential/current, and electrodeposition technique [213].

In the CO_2_RR, the TPB is crucial for efficiently transporting CO_2_ gas to the catalytic surface, thereby improving reaction rates and selectivity [214]. Hydrophobic surfaces can capture CO_2_ gas at the electrode–electrolyte interface, creating a TPB that enhances CO_2_ reduction activity and selectivity [141]. For example, Niu et al. developed a bioinspired Cu catalyst with naturally hydrophobic properties by electrodeposition to mimic the hierarchical structure of Setaria leaves (Figure 8F) [215]. The design alleviated electrolyte flooding during high‐rate CO_2_ electroreduction to generate C_2+_ products, and the engineered Cu structure achieved a high production rate of 255 ± 5.7 mA cm^−2^ with an FE of 64% ± 1.4%, maintaining operational stability at 300 mA cm^−2^ for over 45 h.

Recently, Wang et al. showed that the CO_2_RR performance of Cu catalysts could be significantly improved by in situ electrodeposition (Figure 8G) [216], which exposed and stabilized the Cu(100) facets exhibiting activity for the CO_2_RR. The method increased the surface area, improved selectivity, and achieved an FE of 90% at a partial current density of 520 mA cm^−2^, maintaining constant ethylene selectivity over extended operating periods.

### 2.7 Anode

In CO_2_ electrolyzer systems, the anode complements the CO_2_RR and plays a key role in driving the OER. This reaction is essential for efficiently using the current generated during CO_2_ conversion, but it also contributes significantly to cell voltage increase due to its inherently high overpotential. Therefore, choosing a catalyst that can maintain high activity at low overpotential is crucial to improving CO_2_ electrolyzer efficiency.

In AEM‐based CO_2_ electrolyzers, non‐precious metal catalysts like nickel (Ni) and nickel‐iron (NiFe) composites are commonly used. These catalysts perform well in neutral to mildly alkaline conditions and are cost‐effective and durable. In particular, NiFe composites are gaining attention as essential components for high‐performance CO_2_ conversion systems due to their stable oxygen evolution activity at high current densities. In contrast, iridium oxide (IrO*_x_*) is primarily used in acidic environments required by CEM‐based CO_2_ electrolyzers. Although expensive, IrO*_x_* is highly stable and active in acidic conditions, making it well‐suited for long‐term use.

## 3 Conclusion and Outlook

This review summarizes recent studies on the CO_2_RR to acquire insights into electrolyzer configurations and provide appropriate fabrication‐related guidelines. The electrolyzer components are surveyed, and directions for future development are briefly suggested. Various electrolyzer types, such as H‐type cells, MEA‐type electrolyzers, and flow‐type electrolyzers, are discussed. Additionally, the membranes, electrolytes, flow channels, and PTEs constituting the electrolyzers are introduced. Depending on the phase of the desired product, the selection of an appropriate electrolyzer type and configuring it with optimal components are critical. Therefore, the current challenges, several state‐of‐the‐art technologies, and the most promising solutions are explored.

### 3.1 Need for AEM Development Optimized for CO_2_RR Conditions

Most AEMs are designed for use in fuel cells or water electrolyzers, in which water exhibits relatively beneficial effects. Therefore, new membranes for the CO_2_RR must be developed. To prevent (bi)carbonate formation, the local pH at the cathode surface should be maintained while OH^−^ ions are appropriately transferred to the anode. Additionally, a membrane that allows control over the amount of water passing from the anode should be developed to prevent excessive transfer that could lead to flooding [217, 218].

Sun et al. were able to get hints of membrane fabrication for selective ion transfer from nature [219]. They applied watermelon skin to electrolytic reactions and suggested future directions. Their experimental results revealed that the carboxyl groups in pectin are negatively charged and thus repel negatively charged ions such as HCOO^–^ and CH3COO^–^. However, OH^–^ showed smooth migration. Although the many hydroxyl groups in the watermelon skin membrane system bind with OH^–^ and acid ions through hydrogen bonding, OH^–^ could be transferred through the continuous hydrogen‐bonding networks, whereas acid ions could not.

### 3.2 Focusing on Product Concentration and Yield Over SPCE

With advances in catalyst research and the realization of methods achieving ≈100% FE with current densities of about 1 A cm^−2^, increased attention is being paid to improving the SPCE [109], which reflects the conversion of the input CO_2_ into products. A high SPCE is generally desirable in chemical processes because it reduces the need for downstream product separation, thereby leading to concentrated products and lower energy requirements for recycling unreacted reactants [220]. However, in low‐temperature CO_2_ electrolyzers, maximizing the SPCE often results in low product concentrations and significant HER activity, leading to a lower overall product concentration [110, 112, 221]. This outcome occurs because the SPCE is typically maximized by restricting CO_2_ flow or using acidic cathode environments, neither of which is optimal for generating concentrated CO_2_RR products [79, 222, 223]. Therefore, the product concentration, rather than the SPCE, should be targeted as a meaningful metric for analyzing the CO_2_ electrolyzer performance. Da Cunha et al. recommended that researchers report the product mole fractions and outlet gas compositions to better address the separation demands and process feasibility [224]. To commercialize CO_2_ electrolysis, high product yields and low cell voltages must be prioritized over the SPCE.

### 3.3 Electrolyzer Design for Low‐Concentration CO_2_ Utilization and Byproduct From Combustion

In real‐world applications, CO_2_ is captured from industrial gases or atmospheric air, where it exists at considerably lower concentrations. Notably, flue gas—an industrial combustion product—is mixed with CO at low concentrations of ≈10%–25% [225]. Studies should be conducted to enable the CO_2_RR in a dilute environment by directly utilizing industrial combustion products without a capture process. For instance, Liu et al. recently used LiOH electrolyte to directly capture and utilize atmospheric CO [226]. Based on these early‐stage studies, appropriate catalysts and electrolyzer structures should be developed. Additionally, various byproducts such as SO*_x_* and NO*_x_* are generated upon combustion. When the CO_2_RR is conducted in the presence of combustion products such as SO_x_, NO_x_, and PO_x_, the S–, N–, and P–based byproducts are typically produced, necessitating research on their utilization. Recently, C–P coupling has also been reported in addition to C–C coupling, C–N coupling, and C–S coupling [227, 228, 229, 230].

### 3.4 Increasing Exposure of Specific Crystal Facets via In Situ Electrodeposition

Electrodeposition produces catalysts with diverse morphologies and large surface areas. Simultaneous electrodeposition during the CO_2_RR in a flow cell offers several advantages. The relationship between the intermediates and crystal facets suggests that the intermediates formed during the CO_2_RR can induce and stabilize the formation of specific crystal facets. Intermediates such as *CO, *COOH, and *CO_2_ strongly interact with the Cu(100) facet, promoting the exposure of Cu(100) [216, 231]. This facet is favorable for C–C coupling, whereas the Cu(111) facet favors the formation of single‐carbon products. Therefore, increasing the exposure of the Cu(100) facet through electrodeposition during the CO_2_RR enhances the selectivity toward C_2+_ products and improves reaction efficiency [232]. This approach minimizes electrode reconstruction, maintains electrode stability, and enhances performance at high current densities [194]. Furthermore, this concept could be applied to the electrosynthesis of C–N, C–S, and C–P products using CO_2_.

### 3.5 Challenges in Anodic Reactions to Enhance CO_2_RR Efficiency

In AEM–based CO_2_RR systems, CO_2_ crossover remains a major efficiency barrier [221]. During CO_2_ reduction, bicarbonate and carbonate ions migrate to the anode, where they revert to CO_2_ and escape, reducing system efficiency and complicating CO_2_ recycling. To address this, using potassium bicarbonate (KHCO_3_) as an anolyte has proven effective. KHCO_3_ helps maintain a near‐neutral pH at the anode and acts as a buffer, reducing the rate at which CO_2_ converts to bicarbonates at the cathode [233, 234, 235]. In such reaction environments, IrO*_x_* catalysts are often used for their high OER efficiency and durability, as IrO*_x_* remains stable in near‐neutral conditions. However, the high cost of Ir highlights the need for non‐precious metal alternatives. Additionally, exploring alternative anodic reactions with lower overpotentials than OER offers a promising route to improve system efficiency [236]. Reactions like the hydrogen oxidation reaction [162] and glycerol oxidation reaction [237] operate at much lower overpotentials, helping to reduce cell voltage.

Various methods are being developed to enhance the CO_2_RR electrolyzer performance. By appropriately adopting the research approaches used for electrolyzers in other advanced electrochemical reactions, the significant performance gaps must be bridged to realize commercially viable CO_2_RR systems.

## Conflicts of Interest

The authors declare no conflicts of interest.
